# Detecting T cell receptors involved in immune responses from single repertoire snapshots

**DOI:** 10.1371/journal.pbio.3000314

**Published:** 2019-06-13

**Authors:** Mikhail V. Pogorelyy, Anastasia A. Minervina, Mikhail Shugay, Dmitriy M. Chudakov, Yuri B. Lebedev, Thierry Mora, Aleksandra M. Walczak

**Affiliations:** 1 Shemyakin-Ovchinnikov Institute of Bioorganic Chemistry, Moscow, Russia; 2 Pirogov Russian National Research Medical University, Moscow, Russia; 3 Privolzhsky Research Medical University, Nizhny Novgorod, Russia; 4 Center of Life Sciences, Skoltech, Moscow, Russia; 5 Masaryk University, Central European Institute of Technology, Brno, Czech Republic; 6 Moscow State University, Moscow, Russia; 7 Laboratoire de physique statistique, CNRS, Sorbonne Université, Université Paris-Diderot, and École normale supérieure (PSL University), Paris, France; 8 Laboratoire de physique théorique, CNRS, Sorbonne Université, Université Paris-Diderot, and École normale supérieure (PSL University), Paris, France; University of Edinburgh, UNITED KINGDOM

## Abstract

Hypervariable T cell receptors (TCRs) play a key role in adaptive immunity, recognizing a vast diversity of pathogen-derived antigens. Our ability to extract clinically relevant information from large high-throughput sequencing of TCR repertoires (RepSeq) data is limited, because little is known about TCR–disease associations. We present Antigen-specific Lymphocyte Identification by Clustering of Expanded sequences (ALICE), a statistical approach that identifies TCR sequences actively involved in current immune responses from a single RepSeq sample and apply it to repertoires of patients with a variety of disorders — patients with autoimmune disease (ankylosing spondylitis [AS]), under cancer immunotherapy, or subject to an acute infection (live yellow fever [YF] vaccine). We validate the method with independent assays. ALICE requires no longitudinal data collection nor large cohorts, and it is directly applicable to most RepSeq datasets. Its results facilitate the identification of TCR variants associated with diseases and conditions, which can be used for diagnostics and rational vaccine design.

## Abstract 2

Hypervariable T-cell receptors (TCRs) recognise a vast diversity of pathogen-derived antigens; this study shows that TCRs responding to ongoing immune challenges can be identified using a statistical null model based on sequence similarity.

## Introduction

A major goal of quantitative immunology is to be able to detect and predict T cell receptor (TCR) specificity from high-throughput sequencing of TCR repertoires (RepSeq) data. Current methods that rely on epitope-specific *in vitro* experiments such as MHC multimer assays [1–4] require knowledge of the individual’s HLA type as well as the presented peptide and do not capture the context of the immune response *in vivo*. Alternatives based on mining public TCRs from large cohorts of patients with a common condition [5–9] are very costly and only capture TCRs specific to widely shared HLA/epitope pairs, ignoring the private response. Another approach is to use longitudinal data to identify responding clonotypes [10], but this requires carefully planned experimental setups with time points taken before the infection, which is not always possible. Antigen-specific Lymphocyte Identification by Clustering of Expanded sequences (ALICE) overcomes these issues by predicting TCR involved in the immune response from single repertoire snapshots of single individuals, using sequence similarity.

**Fig 1:**
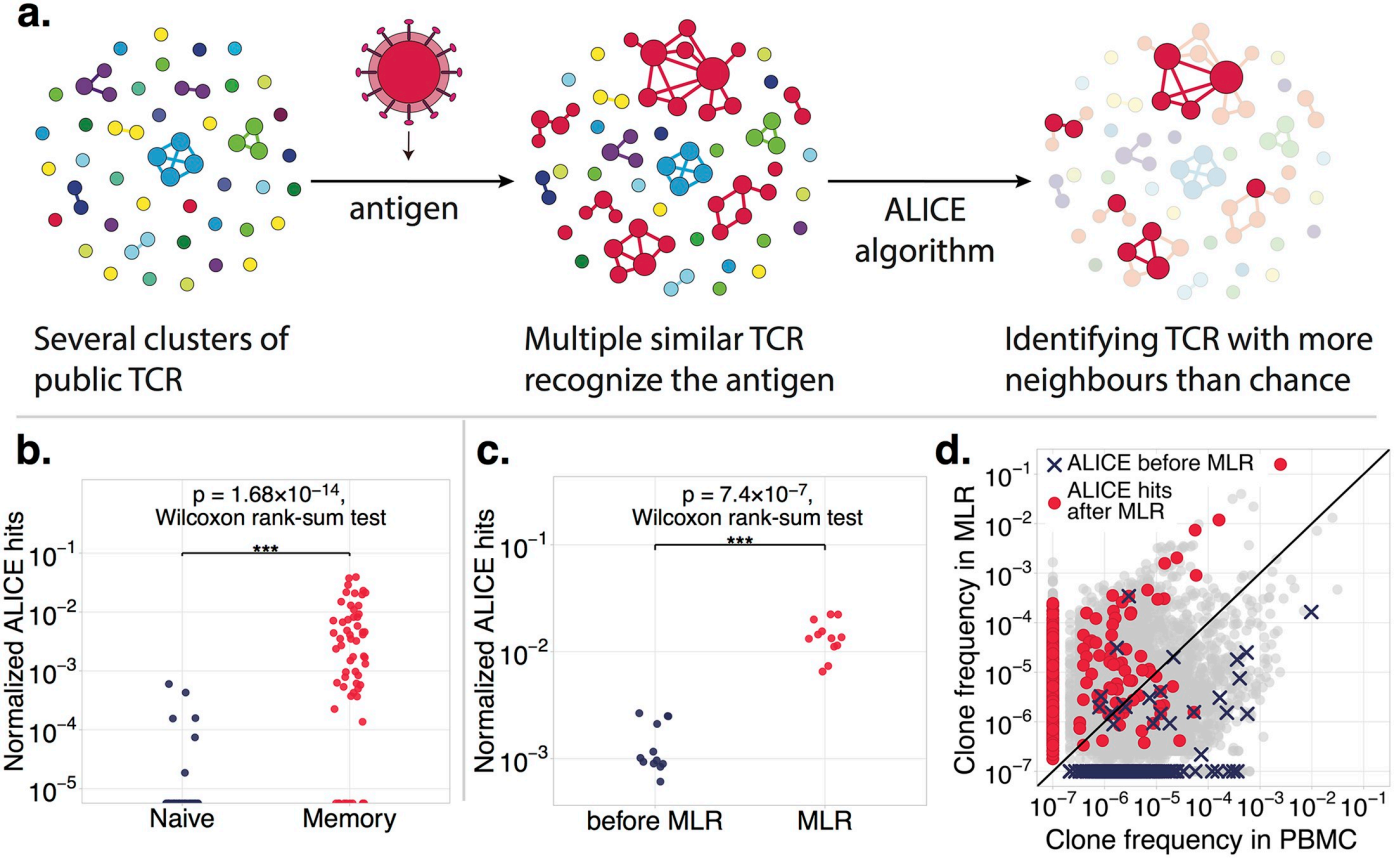
Identification of antigen-responding clonotypes using their recombination-based frequency. (a) ALICE identifies locally enriched regions of the TCR sequence space, represented here as a graph. Vertices are TCR clonotypes observed in the repertoire, and edges connect sequences differing by at most 1 CDR3 amino acid. Antigen exposure induces the proliferation of multiple clonotypes with similar sequences recognizing a few immunodominant epitopes. ALICE identifies clonotypes with a higher numbers of neighbors than expected by a null model of recombination, separating clusters of antigen-responding clonotypes (in red) from clusters arising from recombination statistics (blue, green, and purple clusters). (b) The number of significant results (normalized by the total number of unique nucleotide sequences) found in naive- versus memory-published TCR beta repertoires from Thome and colleagues [16] demonstrates ALICE’s ability to selectively detect immune response signatures in the memory subset only. (c) Normalized number of significant hits found in published repertoires of MLR cultures compared to an unstimulated control [17]. The algorithm finds many more hits in the MLR repertoire. (d) Most clonotypes identified as antigen-responding in MLR culture expanded during the assay, as evidenced by their higher frequency in MLR culture than in the control (red dots). By contrast, clonotypes identified in the unstimulated repertoire (blue crosses) mostly remain unexpanded after the assay, as they probably are signatures of previous immune responses. The individual numerical values could be found in S1 Data. ALICE, Antigen-specific Lymphocyte Identification by Clustering of Expanded sequences; CDR3, Complementarity Determining Region 3; MLR, mixed lymphocyte reaction; PBMC, peripheral blood mononuclear cell; TCR, T cell receptor.

Recent work has shown that TCRs recognizing the same epitopes often have similar sequences [2–4, 11, 12]. However, highly similar TCRs may also arise regardless of their binding properties, by virtue of their high generation probability by V(D)J recombination [13, 14], with clusters of similar TCRs found even in naive repertoires [2, 15]. To correct for those naive clusters, ALICE evaluates the number of similar sequences relative to the baseline expectation from V(D)J recombination statistics, allowing it to identify clusters of TCRs responding to the same antigen (as schematized in Fig 1a).

For each TCR amino acid sequence in the data, ALICE uses a stochastic TCR recombination model [18, 19] to estimate the fraction of the repertoire composed of TCR variants, called ‘neighbors’, differing by at most 1 amino acid in their Complementarity Determining Region 3 (CDR3). This allows us to predict theoretically the number of neighboring clonotypes (nucleotide sequences) for each TCR under the null hypothesis of no antigen-driven TCR selection and identify TCRs with a significantly higher number of neighbors in the data than the null expectation (see Materials and methods). We refer to such significant results as ALICE signatures or hits. Although the basic version of the algorithm discards clonotype abundances and should thus be sensitive to sequencing depth, we also implemented an advanced (but much slower) version that includes read counts and shuffles them among clonotypes in the null (see Materials and methods).

## Results

As a minimal requirement for its validity, we applied our algorithm to published naive (CD45RA^+^CCR7^+^) and effector memory (CD45RA^−^CCR7^−^) TCR beta repertoires from Thome and colleagues [16]. Our algorithm identified multiple signatures in the memory subsets and virtually no significant hits in the naive subsets (Fig 1b and S1 Fig), in agreement with the definition that naive cells have never responded to antigen stimulation.

To further validate the method’s ability to detect clonal expansion during an ongoing immune response, we applied it to published TCR beta repertoires from mixed lymphocyte reaction (MLR) assay [17]. In this assay, peripheral blood mononuclear cells (PBMCs) from 2 individuals (a responder and a stimulator) are mixed, and reactive T-cell clones from the responder’s repertoire proliferate in response to the antigens presented by the stimulator’s cells. ALICE identified many more hits in the responder’s repertoire in the MLR culture than in unstimulated cells (Fig 1c). Furthermore, the clonotypes identified by ALICE are enriched in MLR culture compared to bulk PBMCs (Fig 1d), clearly demonstrating that these hits correspond to antigen-specific clonal expansions.

We then asked whether our method could identify TCRs specific to a particular target using an *in vivo* acute viral infection model. In a previous study, peripheral blood of 6 donors was collected, and their TCR beta repertoire was sequenced at several time points before and after immunization with live yellow fever (YF) vaccine (YF-17D) [10]. Clonotypes that significantly expanded following vaccination were identified by temporal comparisons. Notably, even the most strongly expanded clonotypes after YF immunization are not the most abundant clones in the repertoire even at the peak of the response (day 15 time point), and the overall clone size distribution on day 15 is similar to the one observed before vaccination. Thus, it is not possible to identify expanded clonotypes using only their frequencies on day 15. Here, we applied ALICE to each time point to identify responding clonotypes independently, using only single repertoire snapshots.

**Fig 2:**
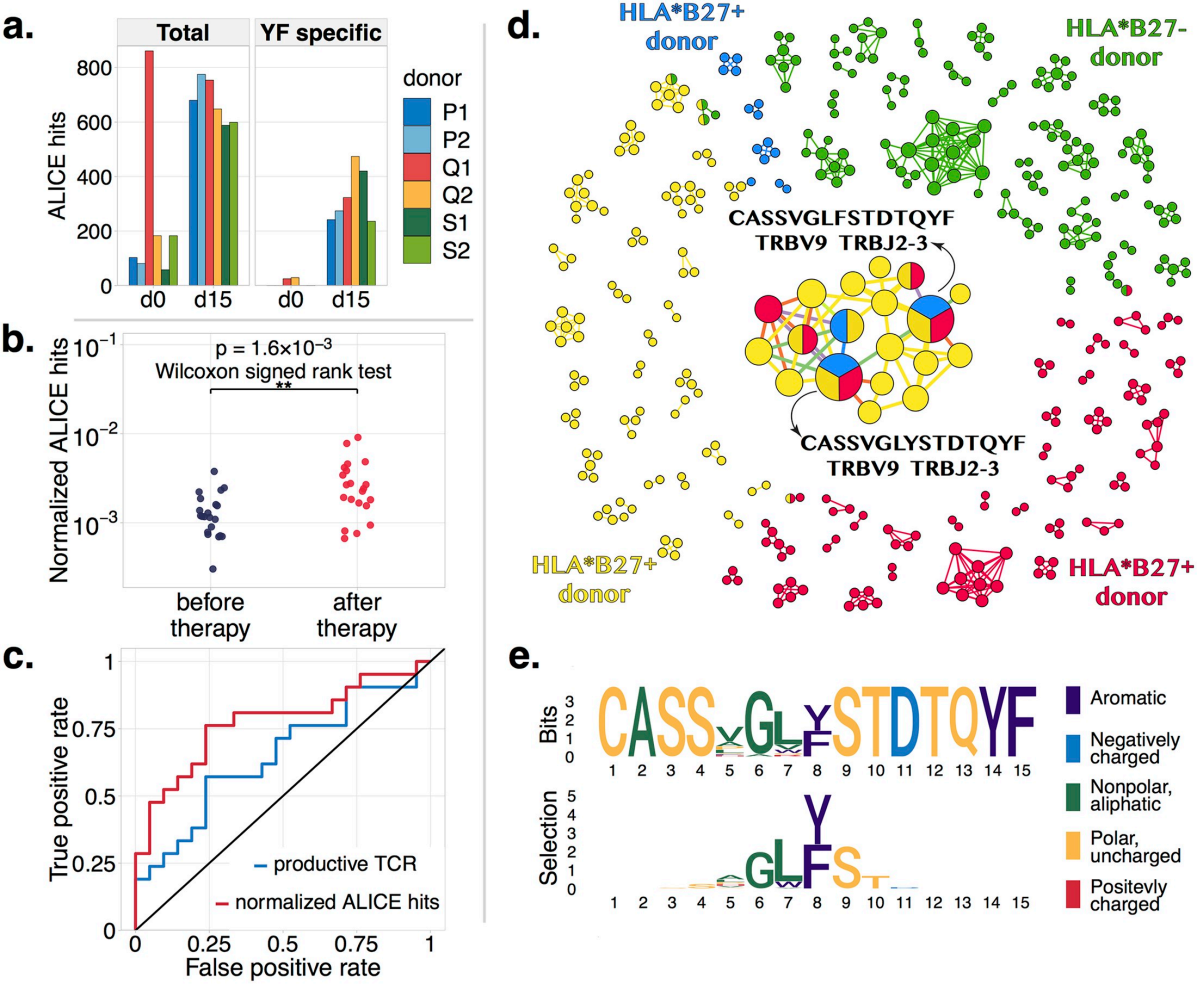
ALICE hits are found in various conditions. (a) Identification of reactive clonotypes following immunization. Left panel shows the total number of ALICE immune response signatures before (day 0) and on the peak of the response to YF vaccine (day 15). Right panel shows the number of clonotypes identified by the algorithm that have high similarity to clonotypes significantly expanded after YF immunization from Pogorelyy and colleagues [10]. (b) Analysis of peripheral blood repertoires before and after checkpoint blockade cancer immunotherapy [20]. The larger number of ALICE signatures after (red) compared with before (blue) therapy represent clones triggered by the therapy. (c) ROC curves for distinguishing pre- and post-immunotherapy repertoires. The number of ALICE hits (red, AUROC = 0.77) is a better discriminant than the number of unique clonotypes (blue, AUROC = 0.65). The individual numerical values can be found in S1 Data. (d) Graph of expanded clonotypes in synovial fluid of 4 AS patients. Vertices represent significant clonotypes identified by the algorithm, and edges connect clonotypes with at most 1 amino acid mismatch. Zero-degree vertices are not shown. Vertices are colored according to the patients, and split vertices represent public sequences identified in several donors. The 2 sequences shared among all 3 HLA-B27^+^ patients were previously associated with AS and HLA-B27. (e) While the classical sequence logo of the central cluster in panel D is dominated by germline-encoded positions (top), selection factors highlight position-specific pressures acting on the expanded sequences (bottom). ALICE, Antigen-specific Lymphocyte Identification by Clustering of Expanded sequences; AS, ankylosing spondylitis; AUROC, area under the ROC; ROC, receiver operating characteristic; YF, yellow fever.

ALICE identified more immune response signatures on the peak of response (day 15) than before immunization (day 0) in all donors (Fig 2a, left) except one who was probably undergoing another immune response at the moment of immunization (see S2 Fig). Applying the advanced version of ALICE with read counts yielded almost identical results (see S3 Fig). To validate ALICE’s expanded clonotypes, we compared its predictions to known YF-17D–reactive sequences obtained from longitudinal data in the source study. We found that 35% to 73% of ALICE hits on day 15 were highly similar (same VJ combination and up to one CDR3 amino acid mismatch) to previously identified YF-specific clonotypes (Fig 2a, right).

Next, we applied our approach to peripheral blood TCR beta repertoire samples from 2 cytotoxic T-lymphocyte-associated protein 4 (CTLA4) checkpoint blockade cancer immunotherapy studies [20, 21]. We found more ALICE immune response signatures after the treatment than before (Fig 2b and S4 Fig). The number of these signatures is a better measure than previously proposed summary statistics of peripheral TCR repertoires aimed at detecting the effect of immunotherapy (richness and Shannon entropy): it discriminated pre- and post-treatment time points better than the number of unique clonotypes (richness) proposed in Robert and colleagues [20], as evidenced by the comparison of their receiver operating characteristic curves (Fig 2c). ALICE hits increased significantly after immunotherapy, both in the data from Robert and colleagues [20] (*p* = 0.0016 for ALICE versus *p* = 0.0005 for richness and *p* = 0.04 for entropy, Wilcoxon signed rank test) and in the data from Subudhi and colleagues [21] (*p* = 0.0003 for ALICE versus *p* = 0.06 for richness and *p* = 0.6 for entropy).

In contrast to these other measures, ALICE identifies particular clonotypes that are likely to be activated by the therapy. Tracking of such clonotypes in time and in the tumor tissue could provide insights into therapy efficiency and adverse effects.

Lastly, we asked whether ALICE was able to identify condition-associated clonotypes in patients with autoimmune diseases. We analyzed 4 TCR beta repertoires of CD8^+^ T cells from the synovial fluid of ankylosing spondylitis (AS) patients from Komech and colleagues [8]. Fig 2d shows clusters of ALICE-predicted clonotypes in 3 HLA-B27^+^ donors and 1 HLA-B27^−^ donor. Although most predicted TCRs were patient-specific, 2 clonotypes were independently found in all 3 HLA-B27^+^ patients but not in the HLA-B27^−^ patient. These 2 clonotypes exactly coincide with previously reported public clonotypes in a population of HLA-B27^+^ patients with AS [7, 8] and were also found in synovial fluid spectratyping of patients with AS and reactive arthritis [22, 23]. The independent identification of these sequences by ALICE demonstrates the relevance of its predictions, as well as suggests that these public clonotypes actively participate in the immune response in inflamed joints. ALICE also predicts previously unreported patient-specific expanded clonotypes, which population studies cannot detect by design.

To visualize CDR3 sequence motifs identified by the algorithm, we developed a novel approach to highlight differences in amino acid composition relative to the background recombination statistics (similar to [3]), based on a position-weight matrix selection model learned from the TCR sequence subset (as in [24], see Materials and methods). In a classical sequence logo derived from the central cluster of Fig 2d, positions encoded in the germline by V and J segments at the two ends of the CDR3 are very conserved and dominate the logo (Fig 2e, top). By contrast, our selection logo highlights amino acids that are enriched relative to that baseline (Fig 2e, bottom), showing a high enrichment in aromatic (Y and F) residues at CDR3 position 8. We speculate that these residues form contacts with the antigen that are crucial for TCR recognition.

## Discussion

Our method can thus be applied to variety of conditions for which information about HLA type or epitope is not available, from autoimmune disease to infection models. While ALICE has different principles and scope than the computational method of Pogorelyy and colleagues [6], which was designed to analyze the public repertoire of cohorts, the two approaches could be combined to leverage the statistical power of large cohorts with the information from sequence similarity exploited by ALICE.

Our approach has several limitations. It can only identify responding TCR with high enough frequencies. A significant fraction of responding TCR are rare and individual specific [10, 12, 15] and are unlikely to have similar variants and thus to be detected by the algorithm. Extending our method to more refined distance measures (e.g., [3]) could help mitigate this issue. Another limitation is a natural consequence of its main advantage — antigen independence. In individuals with multiple conditions, the algorithm will identify clonotypes associated with all of them, and potentially with memory clones from previous immune responses as well, with no way of telling them apart. Repeating the analysis at different time points (e.g., after clearance of an infection) can help to distinguish TCR associated with each condition. In S2 Fig, we performed such an analysis for the outlying YF vaccinee Q1, who was probably undergoing another transient immune response, and identified ALICE signatures that were truly YF-specific. Finally, while ALICE can reliably detect expanded clonotypes from TCR*β* repertoire, specificity is ultimately determined by the full *αβ* clonotype. ALICE could be extended to analyse *αβ* repertoires by estimating the joint probability of *αβ* recombinations using the OLGA algorithm [25], because the two chains are largely independent in their recombinations [26, 27].

As we have illustrated in our applications, the number of ALICE hits can be indicative of the immune activity. This new repertoire-wide metric could be used in combination or comparison with other metrics such as clonality, diversity, or maximum clone frequency [16, 20, 21] to predict immune status. However, ALICE’s main advance is to be able to identify particular sequences, which can be studied across patients that share a condition to identify publicly responding clones, as we did in the AS example. They could be tracked over time during and after the disease to help design biomarkers for diagnostics and to understand the persistence of immune memory. They could be searched in the repertoires of T-cell subpopulations to gain insight into their immunological function. As more repertoire sequence datasets associated with various conditions are being collected, ALICE could be used to rapidly grow databases of condition-specific TCR, with applications in the diagnostic and treatment of diseases.

## Materials and methods

### ALICE statistical model formulation

The algorithm operates on a dataset of *n* unique nucleotide TCR sequences (clonotypes) with a given VJ combination. The procedure is then applied to all VJ combinations present in the data. Unique nucleotide sequences have corresponding amino acid sequences. The goal is to find outlying sequences that have an abnormal number of nucleotide variants in the data that differ by at most 1 amino acid. The algorithm considers distinct nucleotide sequences as separate entities even if they have identical amino acid sequences, because they originate from different T-cell clones.

For each amino acid sequence *σ*, under the null hypothesis we expect the number of neighbors *d* to be Poisson distributed:

$$P(d|\sigma )\;=\;e^{-\lambda }\frac{\lambda^{d}}{d!},$$ (1)

with mean *λ* = *n*Σ_*σ'* ~ *σ*_ *QP*_*gen*_(*σ'*). The sum is over all possible similar variants *σ'* of *σ*. Here, similarity *σ'* ~ *σ* is defined by having at most 1 amino acid mismatch, but other measures could be used instead. *P*_*gen*_(*σ'*) is the probability to generate a given amino acid sequence *σ'* by V(D)J recombination, and *Q* a rescaling factor accounting for thymic selection [6, 14] that eliminates a fraction 1/*Q* of generated sequences. Its value was set to *Q* = 9.41 as the average over all VJ combinations reported by Pogorelyy and colleagues [6]. There is an option in the algorithm to use separate selection factors for different CDR3 lengths *L* within each VJ class, *Q*_*L*|*VJ*_. In this case, *Q*_*L*|*VJ*_ = *Q* × *R*_*L*|*VJ*_ / Σ_*L'*_*R*_*L'*|*VJ*_*P*_*data*_ (*L'*|*VJ*), where *Q* the same scaling factor as above, *P*_*data*_ (*L*|*VJ*) is the probability of CDR3 length *L* given the VJ combination, and *R*_*L*|*VJ*_ = *P*_*data*_ (*L*|*VJ*)/*P*_*gen*_ (*L*|*VJ*), where *P*_*data*_ (*L*|*VJ*) and *P*_*gen*_ (*L*|*VJ*) are the distribution of CDR3 lengths in each VJ class in the data and in the simulated sequences, respectively. We redid analysis for YF-vaccination datasets with this approach and got very similar results (see S5 Fig).

### Estimating the generation probability of amino acid sequences

We estimated *P*_*gen*_(*σ*) of amino acid sequences by Monte Carlo simulation, as described by Pogorelyy and colleagues [6]. We generated 100 million TCRs with fixed VJ choice *in silico* using the VDJ recombination model from Murugan and colleagues [18]. Sequences were then translated to amino acids, and the overall frequency of each distinct amino acid sequence was estimated by counting. The advantage of Monte Carlo simulations is that it can be done for all sequences of interest simultaneously. The exact computation of *P*_*gen*_ of each sequence of interest by OLGA [25] is available within the ALICE software and may be faster for datasets of moderate sizes. Another option would be to use large number of published datasets [5, 28] and treat the number of occurrences of each TCR sequence of interest in these datasets as a proxy for TCR recombination probability, as implemented into VDJtools [29]. An implementation of the corresponding routine for VDJtools software framework is described at http://vdjtools-doc.readthedocs.io/en/master/annotate.html#calcdegreestats. Note that VDJtools implementation allows setting an arbitrary Levenstein distance threshold for defining neighboring clonotypes. Forcing clonotypes to have the same VJ/V segments or allowing segment mismatches is also optional. The implementation relies on a precompiled control dataset instead of using a generative VDJ rearrangement model; control datasets can be obtained from https://zenodo.org/record/1318986.

### ALICE pipeline

Nucleotide sequences with low numbers of reads may represent erroneous variants of high-frequency clonotypes and thus inflate their neighbor counts and lead to false positives. Here, we counted as neighbors only clonotypes with more than 1 read. To correct for sequencing errors in the germline regions, clonotypes with the same CDR3 nucleotide sequence and V- and J-segments were collapsed. To additionally filter sequencing errors, ALICE hits with Monte Carlo–estimated neighborhood size of 0 were also discarded. For each amino acid sequence *σ* present in the data, we count how many one-mismatch variants are also present in the data, and we denote that number *d*(*σ*). The neighborhood of *σ* includes *σ*, meaning that different nucleotide variants of the same amino acid sequence are counted as valid neighbors. For each *σ* such that *d*(*σ*) > 2, we generate all possible one-mismatch variants *σ' in silico* and calculate their *P*_*gen*_(*σ'*) using Monte Carlo simulations as described above. Finally, we calculate a *p*-value for each *σ* corresponding to the probability that *σ* has no less similar variants in null model than in the data, Σ_*d'* ≥ *d*(*σ*)_ *P*(*d'*|*σ*) using Eq 1. We correct *p*-values for multiple testing using Benjamini-Hochberg (BH) correction and select clonotypes with BH-adjusted *p* < 0.001 as significant results (ALICE hits).

### Number of neighbors *per se* is not enough to reliably identify responding clonotypes

The main innovation of our approach is to use the probability of TCR sequence generation, *P*_*gen*_, to get the null model for the expected number of neighbors for each sequence in the data. We wanted to quantify the improvement our null model provides on top of the initial clustering step. We expect the observed number of neighbors, *d*(*σ*), to grow after an immune challenge due to the expansion of many similar clonotypes. *d*(*σ*) scales with the total number of possible neighbors (equal to *n*, the number of unique clonotypes in a given VJ combination). As a simplest method, which does not use *P*_*gen*_, one could simply select all clones with *d*(*σ*)/*n* above a certain threshold. We performed this analysis for the YF-vaccination dataset (see S1 Data). For each donor we picked a threshold *d*(*σ*)/*n* that selects the same amount of clones on day 15 in each donor as we identify with the ALICE approach, and the same threshold is used for the day 0 time point. For brevity, we call clones with *d*(*σ*)/*n* larger than the threshold *d*-hits. The number of *d*-hits is larger after vaccination than before (with the exception of Q1 donor). On day 0, there are 2 to 6 times more *d*-hits than ALICE hits (e.g., for donor P2, 478 d-hits versus 81 ALICE hits). On day 15, much (1.5–40 times) fewer *d*-hits are similar to known YF clones than ALICE hits. Notably, for one twin pair, P1 and P2, almost all *d*-hits are unrelated to the YF vaccination (e.g., for P2, there are 7 YF-like *d*-hits out of 775 on day 15 versus 274 YF-like ALICE hits). To summarize, the number of neighbors alone is not enough to identify interesting and potentially responding clonotypes, but the ALICE approach substantially improves on this, both decreasing the number of false positives (number of hits pre vaccination) and increasing the true positive rate (fraction of YF-like hits post vaccination).

### Including abundance information

The basic pipeline only takes the occurrence of nucleotide sequences in the sample into account, and not their abundance (read count). To include that information, we replace in the pipeline the number of similar sequences, *d*, by a sum of transformed abundances over these sequences, $s\;=\;\sum_{i\;=\;1}^{d}f(c_{i})$, where *c*_*i*_ is the abundance of the *i*^*th*^ nucleotide variant with similar amino acid sequence. There exist several choices for the transformation *f*. *f*(*c*) = 1 − *δ*_*c*,0_ gives back the basic method, *s* = *d*. *f*(*c*) = *c* corresponds to summing the abundances of all similar variants, whereas *f*(*c*) = *log*(*c*) corresponds to summing their logarithms. To define the null model, we assume that the abundance of each sequence is sampled at random from the distribution of empirical frequencies. Because this distribution followed a power law, we worked on the logarithmic scale, and we picked *f*(*c*) = *log*(*c*). To calculate *P*(*s*|*σ*), under null hypothesis, we use the identity *P*(*s*|*σ*) = Σ_*d*_ *P*(*s*|*d*)*P*(*d*|*σ*), where *P*(*d*|*σ*) was computed as described in the basic pipeline (Eq 1). Then, *P*(*s*|*d*) is obtained as a *d*-fold convolution of *P*_*f*_(*f*), the probability distribution of the transformed abundances *f* (*c*_*i*_). For instance, *P*(*s*|*d* = 1) = *P*_*f*_(*f*), *P*(*s*|*d* = 2) = Σ_*f*_*P*_*f*_(*f*)*P*_*f*_(*s* − *f*) = (*P*_*f*_ * *P*_*f*_) (*s*), etc., so that $P(s|d)\;=\;P_{f}^{\left(*\right)}(s)$. These quantities do not depend on *σ* and are computed just once at the beginning of the procedure from the clonotype abundance distribution. Applying this advanced version of ALICE to the YF data of Fig 2a [10] yielded very similar results (S3 Fig) as the basic method. Although it is slower to implement, the advanced method could still be useful because it is expected to be robust to wide ranges of repertoire sampling depths, while the basic version implicitly relies on many sequences not being captured by the sample.

### Statistics

To compare the normalized number of ALICE hits and maximum frequency of productive rearrangement between memory and naive repertoires (Fig 1b), we used Wilcoxon rank-sum two-tailed test (*N* = 52 naive subsets and *N* = 60 memory repertoires, *p* = 2.3 × 10^−14^ for maximum frequency of productive rearrangement, and *p* = 1.7 × 10^−14^ for normalized number of ALICE hits). To compare the normalized number of ALICE hits in repertoires before and after MLR (Fig 1c), we used Wilcoxon rank-sum two-tailed test (*N* = 12 pre-MLR PBMC samples, *N* = 12 MLR cultures, *p* = 7.4 × 10^−7^). To compare number of ALICE hits with other statistics between pre- and post-treatment time points for immunotherapy patients, we used Wilcoxon signed-rank two-tailed test: In Fig 2c (*N* = 21 before and *N* = 21 after therapy), the total number of clonotypes gave *p* = 0.0005, Shannon entropy gave *p* = 0.042, and normalized ALICE hits gave *p* = 0.0016. In S4 Fig (*N* = 40 before and *N* = 40 after first dose of therapy), the total number of clonotypes gave *p* = 0.6, Shannon entropy gave *p* = 0.6, and normalized ALICE hits gave *p* = 0.0003.

### Estimating enrichment in certain amino acid position of the TCR motif

To estimate the enrichment of amino acids at specific positions in the set of expanded TCR, we used a position weight matrix model of TCR selection [24]. The sequence enrichment ratio takes a factorized form over amino acid positions, parametrized by selection coefficient *s*_*i*_(*σ*_*i*_), where *σ*_*i*_ denotes the amino acid of sequence *σ* at position *i*. The predicted frequency in the expanded set is then

$$P_{sel}(\sigma )\;=\;\frac{1}{Z}P_{gen}(\sigma )e^{\sum_{1}^{L}s_{i}(\sigma_{i})}$$ (2)

where *Z* a normalization factor.

The *s*_*i*_ parameters were learned by gradient ascent of the likelihood function, to which an *L*_2_ regularization term, −*λ* || *s* ||^2^, was added. Specifically, selection coefficients are updated according to *s*_*i*_(*σ*_*i*_) ← *s*_*i*_(*σ*_*i*_) + *ε*[*P*_*data*_(*σ*_*i*_) − *P*_*sel*_(*σ*_*i*_) − 2*λs*_*i*_(*σ*_*i*_)], where *P*_*sel*_(*σ*_*i*_) is the predicted frequency of given amino acid at position *i* and *P*_*data*_(*σ*_*i*_) is its observed frequency in the data. After each update, all *s*_*i*_(*σ*_*i*_) are shifted by a common additive constant to satisfy following normalization constraint: $\sum_{a}P_{gen}(\sigma_{i})e^{s_{i}(\sigma_{i})}\;=\;1$.

We applied this inference procedure on the 26 sequences forming the central cluster of sequences from AS patients in Fig 2d. *ε* was set to 0.5, and *λ* was set to 0.02. The algorithm was initialized with *s*_*i*_(*σ*_*i*_) = 0. The iterative procedure was repeated until the sum of the squared update difference was lower than 10^−6^. The bottom logo of Fig 2e shows values of *s*_*i*_(*σ*_*i*_) weighted by amino acid frequencies, so that the height of each letter is *P*_*data*_(*σ*_*i*_)*s*_*i*_(*σ*_*i*_).

## Supporting information

S1 Fig ROC curves for classification of memory and naive repertoires using ALICE hits and maximum productive rearrangement frequency as suggested in the paper by Thome and colleagues [16]. The classifier based on ALICE hits has a much higher true positive rate for low (up to 20%) false positive levels, but it could not distinguish naive and memory subpopulations both having 0 hits. To break these ties, we ranked memory and naive subsets with 0 ALICE hits by the maximum frequency of productive rearrangements (combined classifier, purple curve). The AUROCs for these classifiers are 0.89 (ALICE hits-based), 0.92 (maximum productive frequency-based), and 0.95 (combined classifier). ALICE, Antigen-specific Lymphocyte Identification by Clustering of Expanded sequences; AUROC, area under the ROC curve; ROC, receiver operating characteristic.
(TIFF)

S2 Fig Cumulative fraction of repertoire occupied by immune response signatures of donor Q1. One of the limitations of ALICE is inability to distinguish clonotypes specific for multiple conditions happening simultaneously, for instance, between a response to vaccination and a mild viral infection. Neither the signatures identified on day 0 (blue curve) nor the signatures identified on day 15 (red curve) are able to recapitulate the dynamics of the YF vaccine response. However, the subset of day 15 signatures that are absent on day 0 (purple curve) shows a clear YF-specific response with a peak on day 15. The 122 clonotypes found as significant on both day 0 and day 15 are not similar (defined as 1 amino acid mismatch) to any of the responding clonotypes identified by temporal differences [10], further suggesting that they are not YF-specific but instead correspond to another immune response that is already contracting at day 0. ALICE, Antigen-specific Lymphocyte Identification by Clustering of Expanded sequences; YF, yellow fever.
(TIFF)

S3 Fig Number of ALICE hits identified on day 0 and day 15 after YF vaccination using abundance information. The results of this analysis are almost identical to the results of Fig 2a, with many more signatures identified after immunization than before (with the exception of donor Q1) and a large fraction of ALICE hits identified on day 15 having similar sequences to previously identified YF-specific clonotypes from Pogorelyy and colleagues [10]. ALICE, Antigen-specific Lymphocyte Identification by Clustering of Expanded sequences; YF, yellow fever.
(TIFF)

S4 Fig Number of immune response signatures for data from Subudhi and colleagues [21]. (a) The number of ALICE hits is significantly higher after immunotherapy than before. (b) Scatterplot of the normalized number of ALICE hits before and after therapy in each patient; most points are concentrated above the equality line, showing an increase in the number of hits after therapy in most patients. ALICE, Antigen-specific Lymphocyte Identification by Clustering of Expanded sequences.
(TIFF)

S5 Fig Number of ALICE hits identified on day 0 and day 15 after YF vaccination using separate selection coefficients for different CDR3 lengths. The results of this analysis are almost identical to the results of Fig 2a, with many more signatures identified after immunization than before (with the exception of donor Q1) and a large fraction of ALICE hits identified on day 15 having similar sequences to previously identified YF-specific clonotypes from Pogorelyy and colleagues [10]. Antigen-specific Lymphocyte Identification by Clustering of Expanded sequences; CDR3, Complementarity Determining Region 3; YF, yellow fever.
(TIFF)

S6 Fig Number of *d*-hits (clonotypes with normalized number of neighbors exceeding threshold) identified on day 0 and day 15 after YF vaccination. For each donor we set a threshold on normalized number of neighbors for each clone *d*/*n*, so the selected number of clonotypes on day 15 is the same as identified by ALICE, see Fig 2a. Here, we plot the absolute number of clones exceeding this threshold (*d*-hits). Notably, on day 0 the number of *d*-hits is larger than the number of ALICE hits. On the other hand, the fraction of YF-related *d*-hits is lower (reaching almost 0 for donors P1–P2) on day 15 than the same fraction for ALICE hits. ALICE, Antigen-specific Lymphocyte Identification by Clustering of Expanded sequences; YF, yellow fever.
(TIFF)

S1 Data Individual numerical values for the main and supporting information figures. (XLSX)

## Abbreviations

ALICE: Antigen-specific Lymphocyte Identification by Clustering of Expanded sequences
AS: ankylosing spondylitis
BH: Benjamini-Hochberg
CDR3: Complementarity Determining Region 3
CTLA4: cytotoxic T-lymphocyte-associated protein 4
MLR: mixed lymphocyte reaction
PBMC: peripheral blood mononuclear cell
RepSeq: high-throughput sequencing of TCR repertoires
TCR: T cell receptor
YF: yellow fever
