# Supplementary figures and images for: Detecting T cell receptors involved in immune responses from single repertoire snapshots
Source: PLoS Biol. 2019 Jun 13;17(6):e3000314. doi: 10.1371/journal.pbio.3000314 (PMC6592544; doi:10.1371/journal.pbio.3000314)

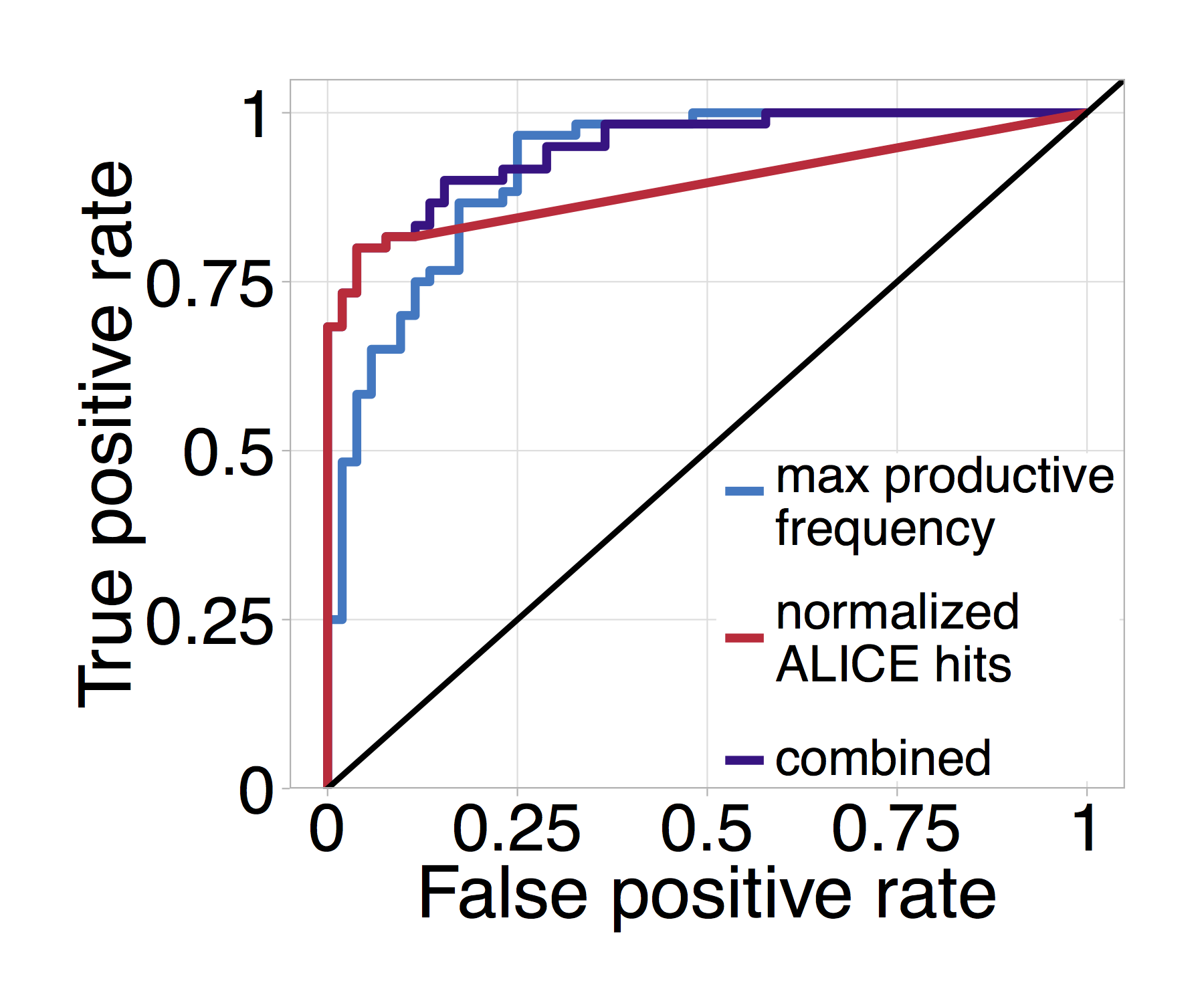

Supplement: S1 Fig — The classifier based on ALICE hits has a much higher true positive rate for low (up to 20%) false positive levels, but it could not distinguish naive and memory subpopulations both having 0 hits. To break these ties, we ranked memory and naive subsets with 0 ALICE hits by the maximum frequency of productive rearrangements (combined classifier, purple curve). The AUROCs for these classifiers are 0.89 (ALICE hits-based), 0.92 (maximum productive frequency-based), and 0.95 (combined classifier). ALICE, Antigen-specific Lymphocyte Identification by Clustering of Expanded sequences; AUROC, area under the ROC curve; ROC, receiver operating characteristic. (TIFF) [file pbio.3000314.s001.tiff]

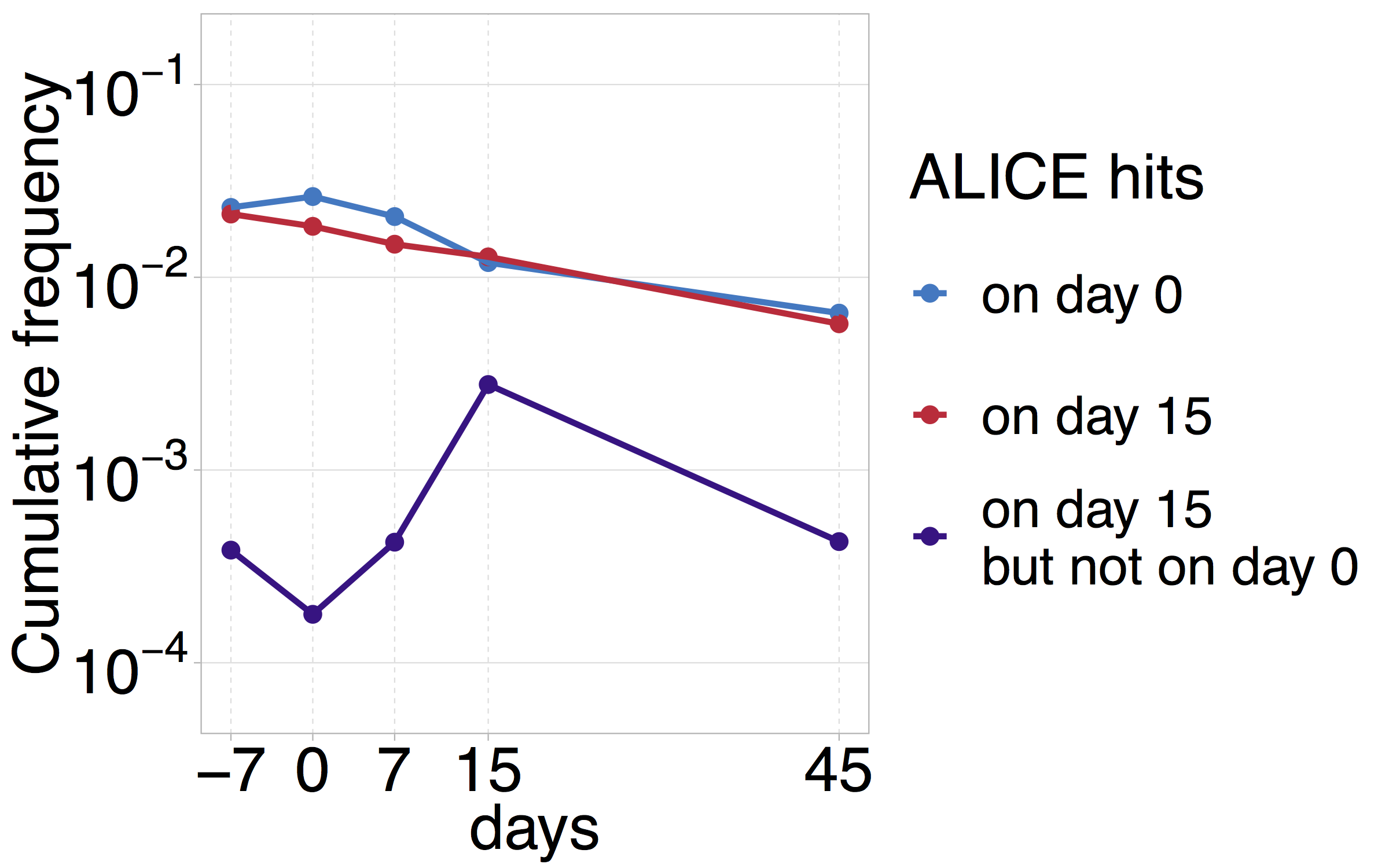

Supplement: S2 Fig — One of the limitations of ALICE is inability to distinguish clonotypes specific for multiple conditions happening simultaneously, for instance, between a response to vaccination and a mild viral infection. Neither the signatures identified on day 0 (blue curve) nor the signatures identified on day 15 (red curve) are able to recapitulate the dynamics of the YF vaccine response. However, the subset of day 15 signatures that are absent on day 0 (purple curve) shows a clear YF-specific response with a peak on day 15. The 122 clonotypes found as significant on both day 0 and day 15 are not similar (defined as 1 amino acid mismatch) to any of the responding clonotypes identified by temporal differences [10], further suggesting that they are not YF-specific but instead correspond to another immune response that is already contracting at day 0. ALICE, Antigen-specific Lymphocyte Identification by Clustering of Expanded sequences; YF, yellow fever. (TIFF) [file pbio.3000314.s002.tiff]

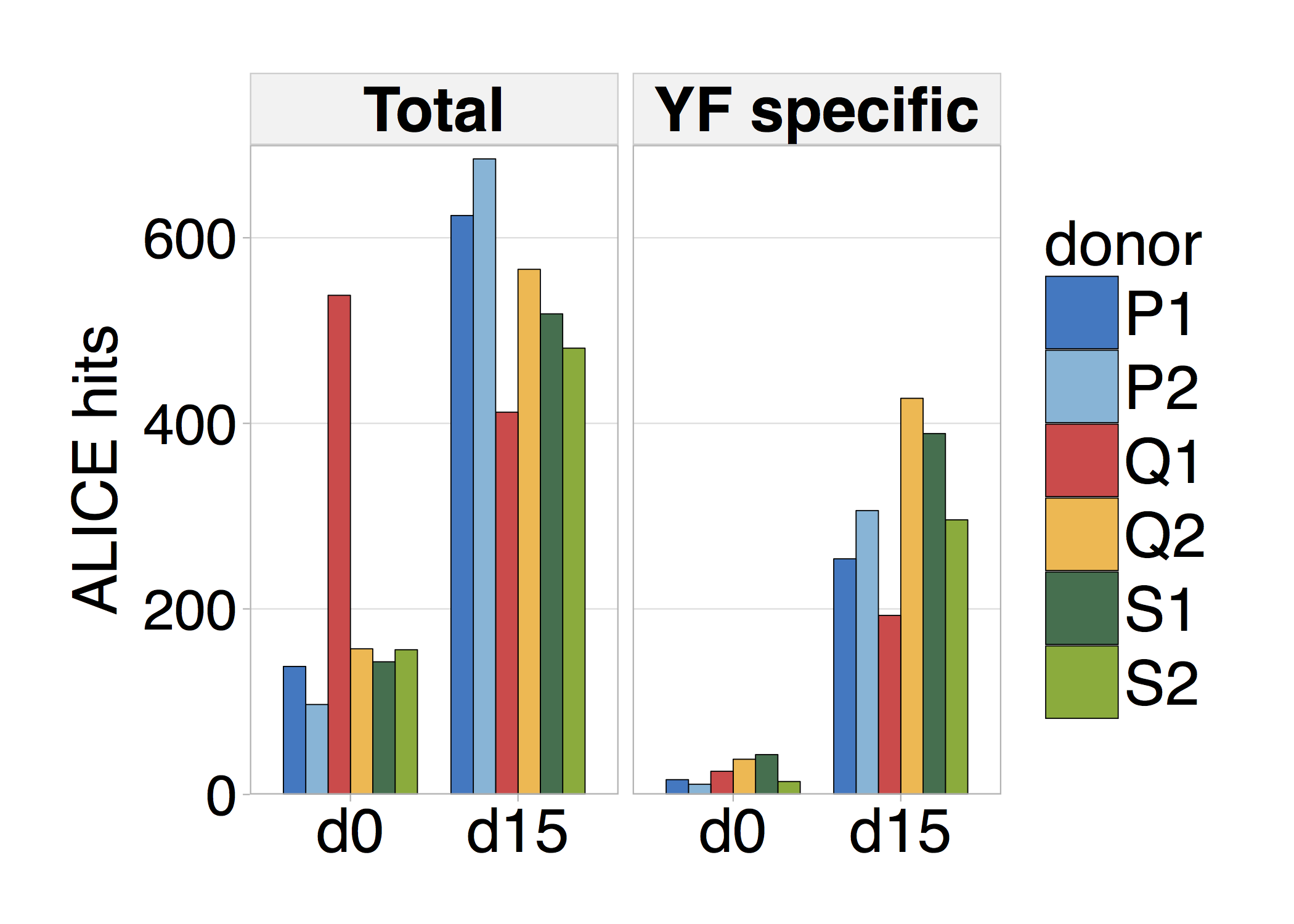

Supplement: S3 Fig — The results of this analysis are almost identical to the results of Fig 2a, with many more signatures identified after immunization than before (with the exception of donor Q1) and a large fraction of ALICE hits identified on day 15 having similar sequences to previously identified YF-specific clonotypes from Pogorelyy and colleagues [10]. ALICE, Antigen-specific Lymphocyte Identification by Clustering of Expanded sequences; YF, yellow fever. (TIFF) [file pbio.3000314.s003.tiff]

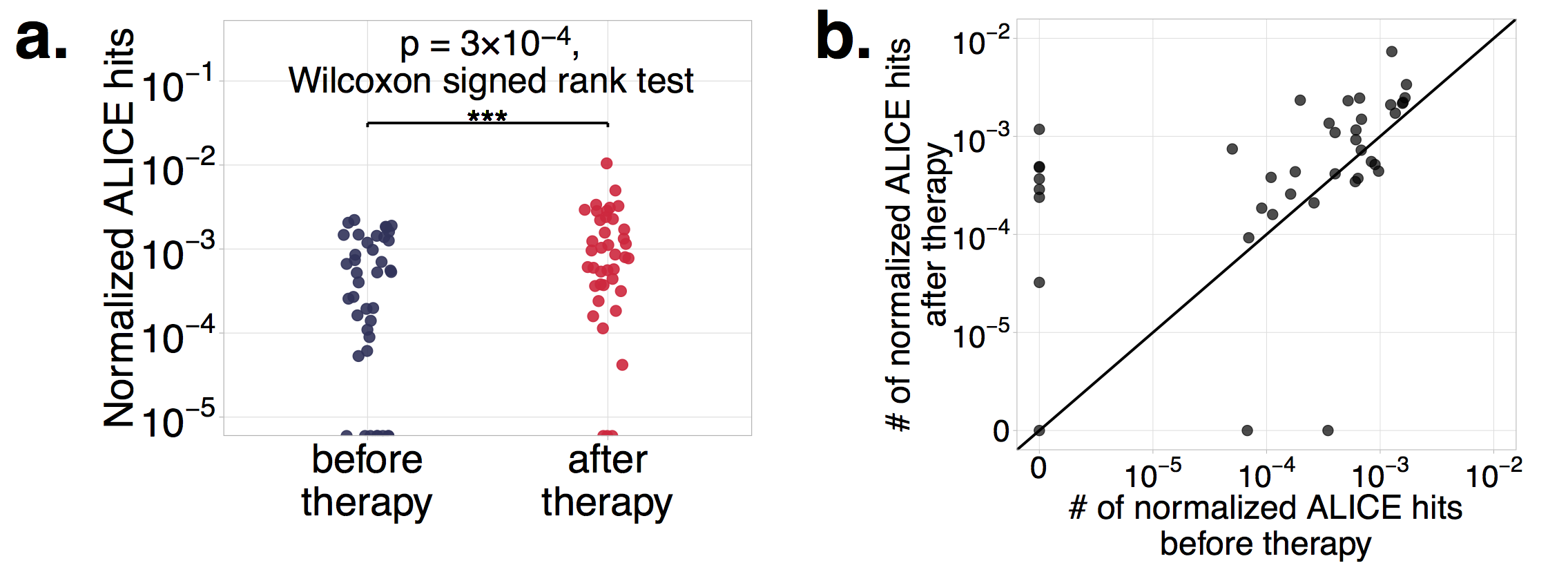

Supplement: S4 Fig — (a) The number of ALICE hits is significantly higher after immunotherapy than before. (b) Scatterplot of the normalized number of ALICE hits before and after therapy in each patient; most points are concentrated above the equality line, showing an increase in the number of hits after therapy in most patients. ALICE, Antigen-specific Lymphocyte Identification by Clustering of Expanded sequences. (TIFF) [file pbio.3000314.s004.tiff]

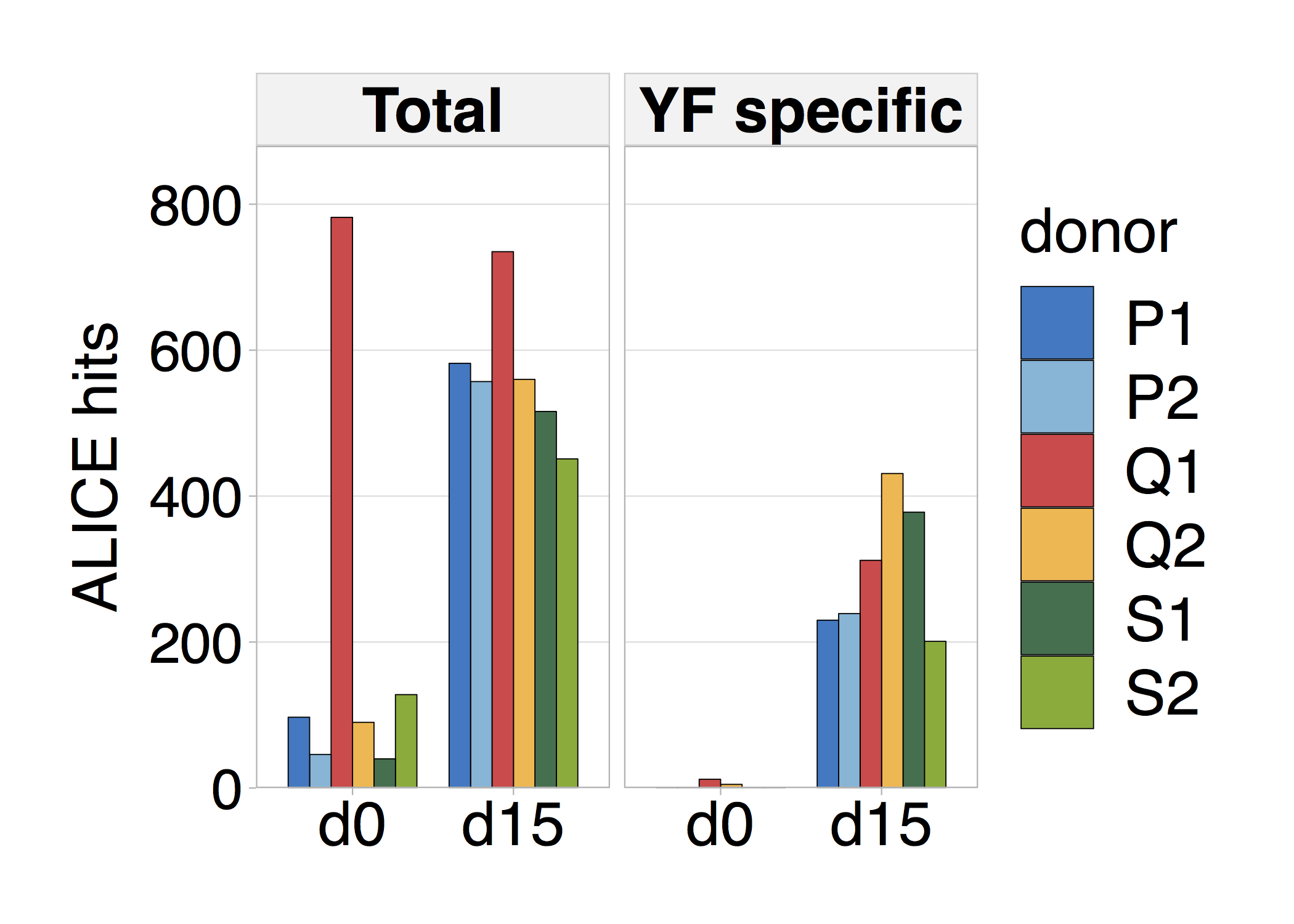

Supplement: S5 Fig — The results of this analysis are almost identical to the results of Fig 2a, with many more signatures identified after immunization than before (with the exception of donor Q1) and a large fraction of ALICE hits identified on day 15 having similar sequences to previously identified YF-specific clonotypes from Pogorelyy and colleagues [10]. Antigen-specific Lymphocyte Identification by Clustering of Expanded sequences; CDR3, Complementarity Determining Region 3; YF, yellow fever. (TIFF) [file pbio.3000314.s005.tiff]

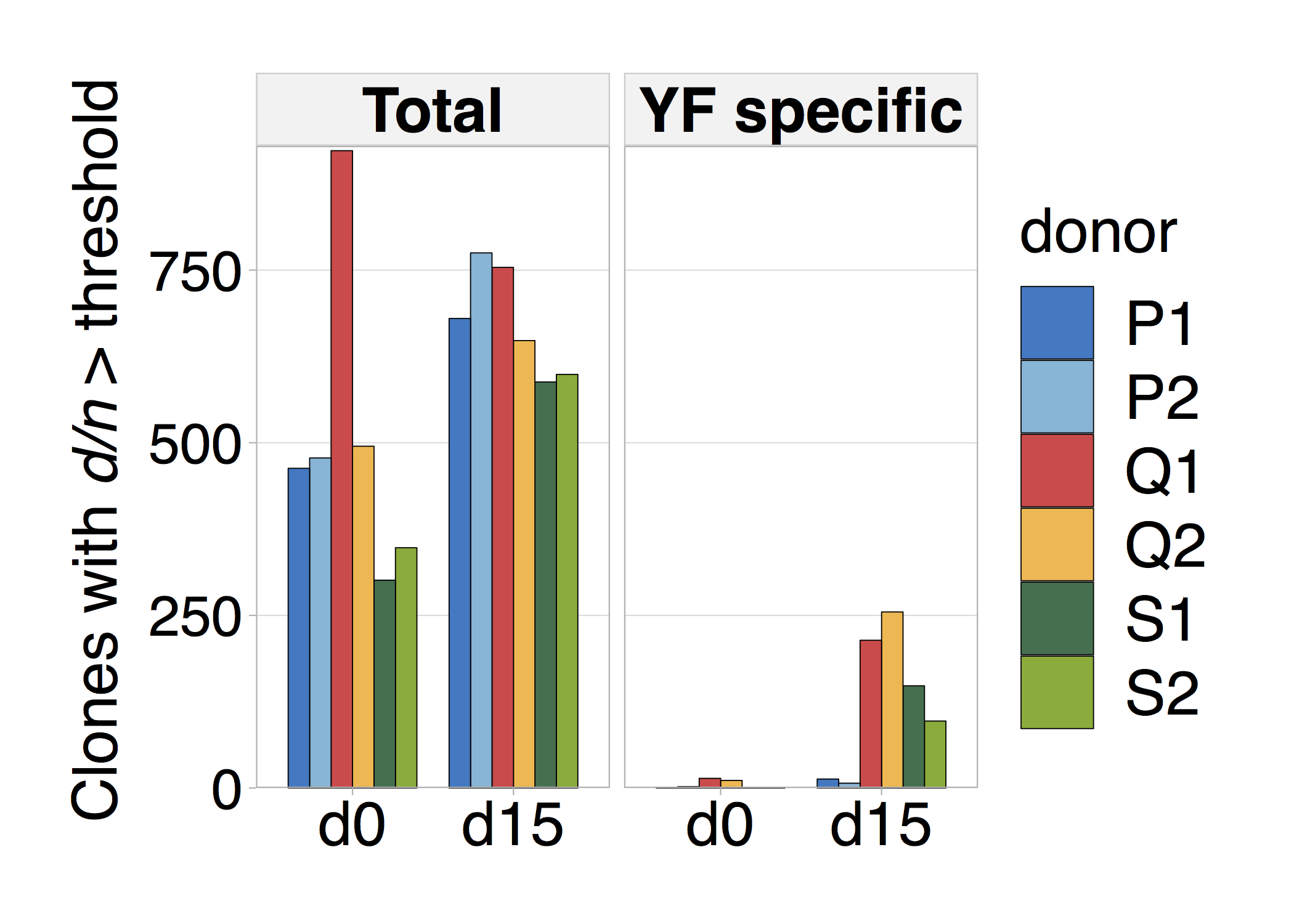

Supplement: S6 Fig — For each donor we set a threshold on normalized number of neighbors for each clone d/n, so the selected number of clonotypes on day 15 is the same as identified by ALICE, see Fig 2a. Here, we plot the absolute number of clones exceeding this threshold (d-hits). Notably, on day 0 the number of d-hits is larger than the number of ALICE hits. On the other hand, the fraction of YF-related d-hits is lower (reaching almost 0 for donors P1–P2) on day 15 than the same fraction for ALICE hits. ALICE, Antigen-specific Lymphocyte Identification by Clustering of Expanded sequences; YF, yellow fever. (TIFF) [file pbio.3000314.s006.tiff]
